# The Impact of Growth Hormone Therapy on Sleep-Related Health Outcomes in Children with Prader–Willi Syndrome: A Review and Clinical Analysis

**DOI:** 10.3390/jcm12175504

**Published:** 2023-08-24

**Authors:** Marco Zaffanello, Angelo Pietrobelli, Giorgio Piacentini, Alessandra Guzzo, Franco Antoniazzi

**Affiliations:** 1 Department of Surgery, Dentistry, Paediatrics and Gynaecology, University of Verona, 37129 Verona, Italy; angelo.pietrobelli@univr.it (A.P.); giorgio.piacentini@univr.it (G.P.); franco.antoniazzi@univr.it (F.A.); 2 Department of Pathology and Diagnostics, School of Medicine, University of Verona, 37129 Verona, Italy; alessandra.guzzo@aovr.veneto.it

**Keywords:** children, polysomnography, Prader–Willi syndrome, quality of life, sleep apnea, sleep-disordered breathing

## Abstract

This literature review of growth hormone (GH) therapy and sleep-related health outcomes in children diagnosed with Prader–Willi syndrome (PWS) assembles evidence for the consequences of sleep deprivation and poor sleep quality: difficulty concentrating and learning at school, behavioral problems, diminished quality of life, and growth impairment. Sleep-disordered breathing (SDB) is another factor that impacts a child’s well-being. We searched the electronic databases Medline PubMed Advanced Search Builder, Scopus, and Web of Science using MeSH terms and text words to retrieve articles on GH deficiency, recombinant human growth hormone (rhGH) therapy, sleep quality, SDB, and PWS in children. The censor date was April 2023. The initial search yielded 351 articles, 23 of which were analyzed for this review. The study findings suggest that while GH may have a role in regulating sleep, the relationship between GH treatment and sleep in patients with PWS is complex and influenced by GH dosage, patient age, and type and severity of respiratory disorders, among other factors. GH therapy can improve lung function, linear growth, and body composition in children with PWS; however, it can also trigger or worsen obstructive sleep apnea or hypoventilation in some. Long-term GH therapy may contribute to adenotonsillar hypertrophy and exacerbate sleep apnea in children with PWS. Finally, GH therapy can improve sleep quality in some patients but it can also cause or worsen SDB in others, leading to diminished sleep quality and overall quality of life. The current evidence suggests that the initial risk of worsening SDB may improve with long-term therapy. In conclusion, rhGH is the standard for managing patients with PWS. Nonetheless, its impact on respiratory function during sleep needs to be thoroughly evaluated. Polysomnography is advisable to assess the need for adenotonsillectomy before initiating rhGH therapy. Close monitoring of sleep disorders in patients with PWS receiving GH therapy is essential to ensure effective and safe treatment.

## 1. Introduction

Sound sleep is vital for physical and mental well-being. During sleep, brain activity is reduced and muscles are relaxed. Sleep is regulated by myriad intricate biological mechanisms, including circadian rhythm, sleep neurons, and neurochemical activity. Circadian rhythm governs the body’s sleep–wake cycle, while sleep neurons regulate the transition between wakefulness and rest. Neurochemical regulation involves various nervous systems and chemical substances working together to maintain sleep balance [1].

Lack of sleep and inadequate rest in children are detrimental to their physical and mental health, resulting in difficulty concentrating and learning at school, irritability, problematic behavior, stunted growth, weakened immune defense, and anxiety and depression. In brief, sleep deprivation has wide-reaching consequences for growth and development, learning, behavior, and well-being [2].

Growth hormone (GH) is produced by the pituitary gland; it promotes cell division and protein synthesis for body growth and development. GH secretion is regulated by a complex feedback system involving the pituitary gland, the hypothalamus, and other organs during deep sleep [3].

Primary growth insufficiency, or hypopituitarism, refers to insufficient GH production by the pituitary gland. This can lead to short stature, delayed puberty, and other symptoms. Primary growth insufficiency is rare, affecting about 1 in 4000 people. Usually diagnosed in childhood, primary growth insufficiency has no cure but can be treated with GH therapy [4]. Diagnosis is based on hormonal and imaging tests; treatment entails synthetic GH replacement therapy administered via subcutaneous injection to stimulate growth [5,6,7]. GH deficiency can also occur in genetic disorders such as Prader–Willi syndrome (PWS), in which GH deficiency is just one of many symptoms [8].

Based on multidisciplinary assessment [9], preferably conducted by specialists, treatment with recombinant human GH (rhGH) should be considered for patients with PWS. While no consensus exists on the exact age to start rhGH therapy, initiating treatment before the age of 2 years is considered beneficial. Furthermore, polysomnography (PSG) should be performed before initiating therapy. Evidence for its efficacy in infants and children is derived from trials in which the daily dose was 1.0 mg/m^2^/day. Exclusion criteria should include severe obesity and untreated severe obstructive sleep apnea (OSA). Patients should be monitored for the potential side effects of rhGH, such as sleep-disordered breathing (SDB) and excessive daytime sleepiness [10].

Sleep-disordered breathing (SDB) is becoming increasingly prevalent among children and adolescents [11,12]. Obstructive SDB refers to a syndrome of upper airway dysfunction during sleep, characterized by snoring and/or increased respiratory effort secondary to upper airway resistance and pharyngeal collapsibility [13]. Pediatric SDB is associated with various complications, including growth deficit [14], metabolic alterations [15], cardiovascular disorders [16], and poor sleep quality, as well as neurocognitive impairments [17], which impact the patient’s quality of life (QoL) [18,19]. Furthermore, children with genetic disorders can benefit from the screening and treatment of obstructive sleep apnea syndrome (OSAS) [20].

### Study Aims

**Figure 1:**
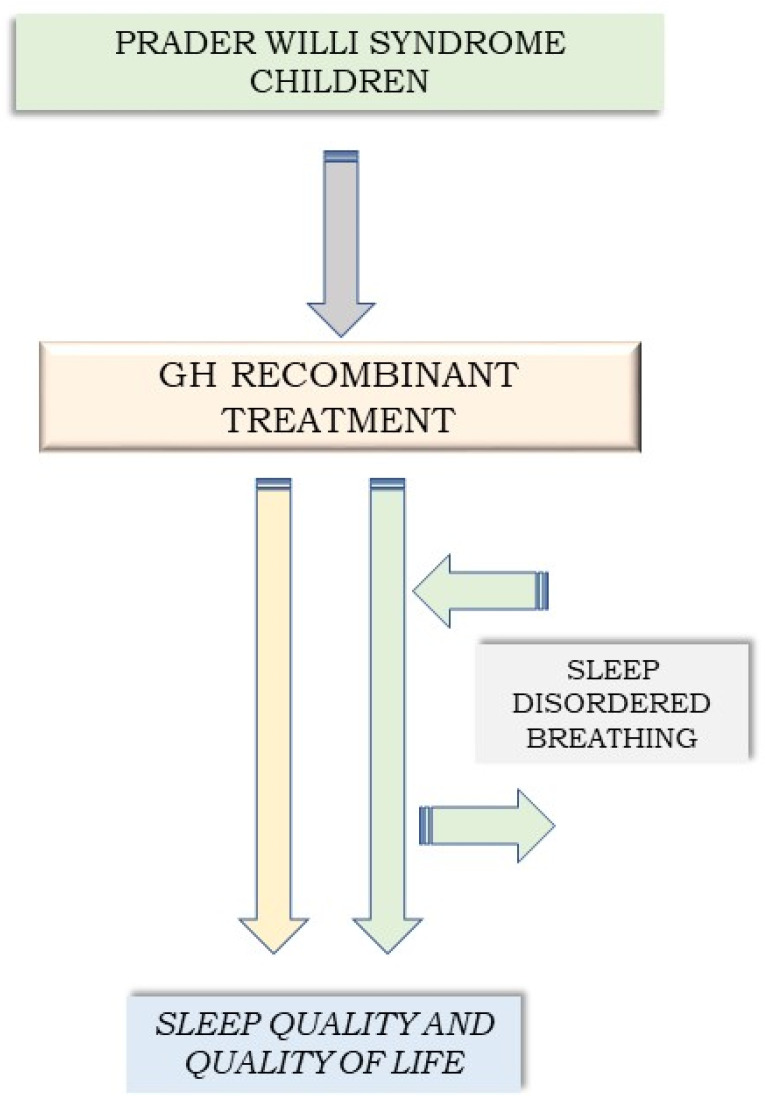
Study flow chart.

**Figure 2:**
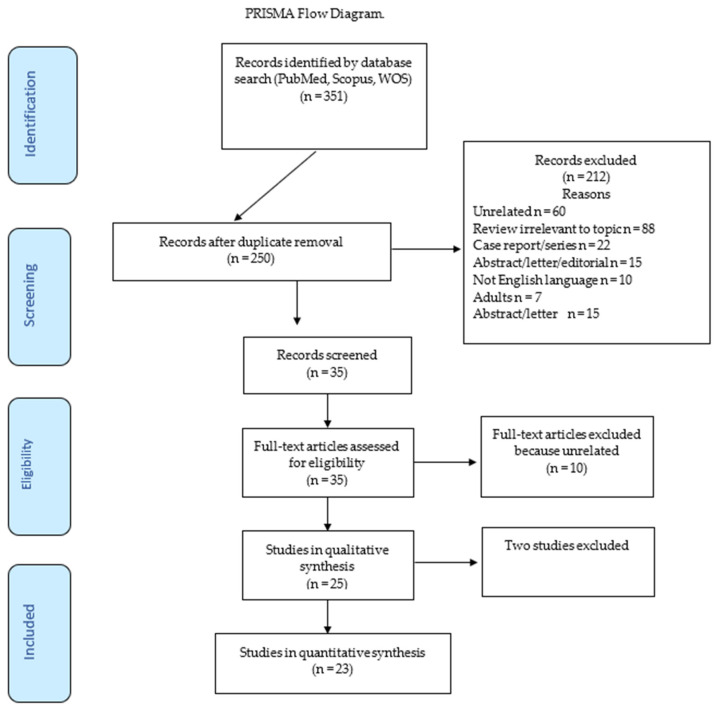
The PRISMA flowchart illustrates the study selection process.

This literature review assembles evidence about the effect of rhGH treatment on sleep in children with PWS and SDB. The aims were: (1) to evaluate studies investigating the effects of GH therapy on SDB in children with PWS (Figure 1), and (2) to provide clinical insight into the risks and benefits of GH therapy in children with PWS.

With this study, we assessed the impact of recombinant human GH (rhGH) therapy on sleep quality and quality of life in patients with PWS and SDB.

## 2. Materials and Methods

We searched the electronic databases Medline PubMed Advanced Search Builder, Scopus, and Web of Science (WOS) using the following MeSH terms (https://meshb.nlm.nih.gov/, access on 5 May 2023) and text words:

(1) (children OR infants OR pediatric) AND (“growth hormone” OR GH OR “GH deficiency” OR “GH treatment”) AND (“sleep quality” OR “sleep pattern” OR “sleep duration and efficiency” OR (rest OR “sleep depth” OR “good sleep quality” OR “sleep satisfaction” OR “sleep health”) AND (“Prader–Willi syndrome”);
(2) (children OR infants OR pediatric) AND (polysomnography OR “nighttime apnea” OR “disrupted breathing during sleep” OR “sleep-related respiratory disorders” OR “obstructive sleep apnea syndrome”) AND (“GH treatment” OR “GH therapy” OR “GH replacement therapy”) AND (“Prader–Willi syndrome”);
(3) (children OR infants OR pediatric) AND (polysomnography OR “nighttime apnea” OR “disrupted breathing during sleep” OR “sleep-related respiratory disorders” OR “obstructive sleep apnea syndrome”) AND (“growth hormone” OR GH OR “GH deficiency” OR “GH treatment”) AND (“Prader–Willi syndrome”).

The censor date was April 2023. Two reviewers independently extracted the data in duplicate from each study to minimize errors and distortions in their interpretation. A third reviewer was involved in resolving any discrepancies among the reviewers, ensuring the accuracy and consistency of the data extraction. Additionally, the reviewers assessed the methodological quality of each study, including the study design robustness and result validity, to evaluate the overall quality of the scientific evidence. The PRISMA flowchart below illustrates the selection process (http://www.prisma-statement.org/PRISMAStatement/FlowDiagram access on 5 May 2023).

## 3. Results

The initial search yielded 351 articles evaluated against the inclusion and exclusion criteria. A total of 35 articles underwent full-text analysis. A total of 10 were excluded, and 25 underwent in-depth evaluation. Two were excluded after collegial discussion to resolve disagreement among the reviewers (Figure 2). The first study [21] reported that while GH therapy may rarely lead to OA in children, GH administration increased the occurrence of OA in only a subset of patients. The second study [22] stated that OSAHS can lead to growth retardation in children and that adenotonsillectomy can effectively treat growth retardation associated with OSAHS. Following adenotonsillectomy, insulin-like growth factor 1 (IGF-1) serum levels were increased, indicating growth. Also, the study suggested that OSAHS may impact GH levels in children. Overall, the two studies suggest that adenotonsillectomy may be beneficial for reversing growth retardation associated with OSAHS, while monitoring IGF-1 levels can help assess growth response. Finally, 23 articles were found to be relevant to this study’s aims. Table 1 presents the study characteristics and summarizes the findings for rhGH therapy in SDB. 

Several studies suggested an association between rhGH therapy and OSA [23,24] in children with PWS. Since the studies involved different patient populations and employed diverse methods to assess OSA or differed in length, it is difficult to compare the results. 

Haqq et al. [25], Scheermeyer et al. [26], and DeMarcantonio et al. [27] an improvement in the apnea–hypopnea index (AHI) and oxygen saturation following GH therapy. Salvatoni et al. [28] stated that short-term GH therapy did not cause upper-airway obstruction in patients with PWS and did not increase obstructive AHI. Miller et al. [29] found that GH treatment did not substantially impact SDB in children with PWS.

OSA in children with PWS appears to develop independently of treatment initiation. Williams et al. [30] found that GH therapy influenced the BMI z-score but did not affect the presence or severity of SDB. Festen et al. [31] observed no worsening of SDB during 6 months of GH treatment. Zimmerman et al. [8] found no significant differences in the respiratory parameters based on patient age at treatment initiation. Schaefer et al. [32] found a reduction in the severity of respiratory events in children with PWS during GH treatment. Tan et al. [33] stated that respiratory parameters did not significantly change with rhGH treatment in children with PWS.

Miller et al. [34] found an improvement in AHI with GH therapy, but 32% of patients experienced worsening SDB during GH treatment. Katz-Salamon et al. [35] stated that SDB is highly common in patients with PWS and that GH therapy does not significantly improve respiratory function. According to Caudri et al. [36], GH therapy did not cause marked changes in the frequency of respiratory events, but 13% of patients developed moderate/severe OSAS.

Khayat et al. [37] reported that GH therapy may worsen OSA in patients with PWS and adenotonsillar hypertrophy. Pavone et al. [38] observed that GH treatment may require upper-airway surgery or non-invasive ventilation in some patients; they also found an increase in the oxygen desaturation index (ODI) in patients who started rhGH therapy, but no significant differences between the group receiving rhGH treatment and the group that did not. Meinhardt et al. [39] recorded three cases of sleep apnea during long-term GH treatment, with no other serious adverse events reported. Lecka-Ambroziak et al. [40] stated that rhGH treatment can influence the severity of SDB in children with simple obesity and PWS. The mortality risk appears to be higher in children with PWS on GH treatment, especially in the first 9 months of therapy [41].

**Table 1:** Studies investigating the relationship between growth hormone (GH) therapy and sleep in children with Prader–Willi syndrome.

Author	Year Published	Country	Study Design	PWS Patients—No.	Age	Methods	SDB Severity	rhGH	Outcome	Conclusion
Haqq et al.	2003	USA	RCT	14 (7 males)	4.5–14.5 yrs	rhGH therapy (or placebo for 6 mths, then alternative intervention for 6 mths), PSG (at 0, 6, and 12 mths)	PWS vs. normals: apnea events 50.6 ± 69.0/h vs. 27.3 ± 20.4/h (*p* = 0.26); hypopneic events 146 ± 55/h vs. 114 ± 65/h, *p* = 0.18	0.043 mg/kg/day in 6 patients	rhGH therapy improved sleep, behavior, cognition	Hypopnea and apnea improved after rhGH therapy
Miller et al. [34]	2006	USA	Longitudinal	25 (15 males)	6 mths to 39 yrs	rhGH therapy (baseline and after 6 wks), PSG (repeat PSG after 6 mths of GH therapy)	GH improved AHI by mean of 1.2/h (*p* = 0.02); CA by median of 1.7/h (*p* < 0.001)	0.24 mg/kg/wk	AHI improved after rhGH therapy; OA worsened in 6 patients (3 males)	AHI improved after rhGH therapy; initial transient worsening of SDB in patient subset
Festen et al. [31]	2006	Netherlands	RCT	53 prepubertal (30 males)	Median of 5.4 yrs (IQR 2.1–7.2)	rhGH therapy, PSG (repeat PSG after 6 mths of GH therapy) in 39 patients	AHI 5.1 (2.8–8.7)/h; CA 2.8 (1.5–5.4)/h	Somatropin 1 mg/m^2^/day	No differences in SDB and SpO_2_ before and after 6 mths of rhGH therapy; AHI decreased after 6 mths of rhGH therapy	No worsening of SDB during rhGH therapy
Craig et al. [41]	2006	Australia	Prospective	328 (83.5% prepubertal)	Median of 6.0 yrs (prepubertal median 12.7 yrs)	rhGH therapy, PSG (repeat PSG after 6 mths of GH therapy)	Case 1 died following presumed SA 3 mths after rhGH initiated	0.23 (0.15–0.31) mg/kg/wk in prepubertal and 0.22 (0.12–0.30) mg/kg/wk in pubertal children	Sudden death (bronchopneumonia, respiratory failure, SA) in 5/675 cases	GH therapy to be used with caution in patients with extreme obesity or SDB
Williams et al. [30]	2008	USA	Observational	37 (54% males)	9 ± 6 (range of 15 mths to 24 yrs)	rhGH therapy, PSG, Multiple Sleep Latency Test (MSLT)	Mean AHI of 17/h (*n* = 37); mean CA of 1.7/h (*n* = 37)	Mean of 0.22 mg/kg/wk (*n* = 16), treated for 4.8 yrs (range of 1.5–12)	All patients had SA; no difference in AHI, CA, SpO_2_ between the rhGH and the non-rhGH groups	rhGH therapy did not affect SDBs
Miller et al. [29]	2009	USA	Pilot	20 infants (12 males)	2–21 mths	rhGH therapy, PSG (repeat sleep studies at 6 wks after GH therapy initiated)	Pre-OA: median of 35.8/h; post-OA: 34.8/h; pre-CA: median of 25.2/h; post-CA: 27.1/h	Start 1 mg/m^2^/day	rhGH therapy did not affect SDBs; increased OA associated with respiratory infections or GER in 12 children after rhGH therapy	rhGH therapy did not affect SDBs
Fillion et al. [24]	2009	Canada	Retrospective	23 (14 males)	8.6 yrs (range of 1.3–13.5) in GH group; 5.0 yrs (range of 2.0–13.0) in no-GH group (*p* = 0.43)	rhGH therapy (10 patients treated for 0.1 to 5.5 yrs), PSG (patients with signs and symptoms of OSA)	2/10 died, 1 developed OSA 2 mths after starting rhGH; OSA disappeared after GH discontinued	0.25 mg/kg/wk (range of 0.14 to 0.42)	rhGH associated with OSA	Did not directly evaluate correlation between GH therapy and sleep
Salvatoni et al. [28]	2009	Italy	Longitudinal observational	34 non-severely obese (20 males) (children with OA and/or severe obesity excluded)	0.94–11.8 yrs; median of 2.24	rhGH therapy, PSG, ENT evaluation	OAHI increased in 8/16 (50%) children and decreased in 5/16 (31%)	0.03 mg/kg/day	AHI increased in 50% of patients (not significant) after 6 wks of rhGH therapy; rhGH did not cause upper-airway obstruction	Short-term rhGH therapy did not cause upper airway obstruction. No increase in AHI in patients receiving rhGH vs. controls
DeMarcantonio et al. [27]	2010	USA	Retrospective	5	Median of 5.1 yrs (range of 1.1–16.7)	OSA surgery; effect of rhGH therapy on OSA; PSG (post-operative PSG performed at median of 8.7 mths (range of 1.75–33.3) after surgery	Median AHI decreased from 16.4/h to 4.4/h (*p* = 0.274)	NA, (3 patients received therapy and 2 received treatment prior to surgery)	Complete resolution of OSA challenging to achieve with upper-airway surgery	PSG evaluation for OSA in children considered for rhGH therapy
Meyer et al. [42]	2012	USA	Comparative	13 (7 males)	rhGH therapy initiated at median of 8.5 mths (range of 2 mths to 6 yrs)	rhGH, A&T, PSG (PSG repeated if upper-airway surgery performed)	9/13 patients with mild to moderate OSA; breathing normalized after A&T in 8/9	NA	Increase in CAs may occur; 2 children with highest postoperative AHI aged 2 yrs and 6 mths, respectively, had initiated rhGH at age 4 and 2 mths, respectively	Starting rhGH therapy at younger age may have contributed to development of OSA at younger age
Katz-Salamon et al. [35]	2012	Sweden	Clinical case series	16 (7 males)	rhGH initiated at median age of 30 mths (range of 5–63 mths),	rhGH therapy follow-up at 6 mths (range of 2–32) after initiating GH, basal PSG	Before GH treatment: AHI (h): 1.32 (0.3–2.8)/h; On GH treatment: AHI: 0.8 (0.1–26)/h, *p* = 0.06	NA (start at median age 30 mths (range of 5–63)	Gender, age at initiating rhGH therapy, and duration did not influence cardio-respiratory responsiveness to CO_2_/O_2_	rhGH therapy improved circulatory stress and function; saturation in GH-treated children better, although AHI remained unchanged
Berini et al. [43]	2013	Italy	Observational	75	1.9 yrs, IQR 2.2 (0.4–7.8)	rhGH therapy, PSG (prior to treatment, up to 4 yrs)	AHI > 1 during treatment in 11/50 (22%) patients	0.010 to 0.030 mg/kg/day	AHI improved with progressive decrease in CAI during rhGH therapy; therapy temporarily discontinued in 3 patients	rhGH therapy did not impair respiratory function during sleep
Vandeleur et al. [23]	2013	Australia	Observational	34 prepubertal children (17 males)	Mean of 7.3 yrs (range of 3 mths–16.3 yrs)	PSG prior to rhGH therapy	OSA in 15/34 (44%) patients	NA	CAI > 5/h in 4/34 children (range of 0.7–15.6 yrs); rhGH therapy deferred in 38%	OSA diagnosed in 44% before starting GH therapy; more likely in older children
Meinhardt et al. [39]	2013	Switzerland	Clinical trial	41 prepubertal children	Mean of 3.8 ± 3.0 yrs	Medical records of children receiving rhGH therapy for over 12 mths, (PSG not stated)	SA recorded in 3 (7.3%) patients	0.03–0.06 mg/kg/day for 4.1 yrs (range of 0.9–9.5)	Apnea recorded in 1/41 patients on long-term rhGH	No serious adverse effects reported
Cohen et al. [44]	2014	Canada	Observational	44 (20 males)	Median of 1.9 yrs (range of 0.3–15.6)	rhGH therapy PSG	Median CAI: 10.6 (range of 5.0–68.3)/h. Median oAHI in those with OSA: 4.0 (range of 1.5–57.0)/h); OSA predominated in older children (52% vs. 5% *p* = 0.001)	NA	CA more likely to occur in infants; OA more prevalent than CA in older children	Most common indication for referral was evaluation prior to initiation of GH therapy
Pavone et al. [38]	2015	Italy	Multicentric	88 (44 males)	Median of 5.1 yrs (range of 0.3–44.3) (IQR 1.0–14.5)	rhGH therapy, overnight respiratory polygraphy (before GH initiated)	Mixed AHI of 1.8 [0.6–5.0]/h, and CAI of 0.1 [0.0–0.6]/h	NA	PGs performed in routine care before initiating rhGH therapy; rhGH started in 48 patients	High prevalence of SDB and number of therapeutic interventions after PG; aim of rhGH therapy to improve natural course of disease
Khayat et al. [37]	2017	Canada	Observational	28 infants (12 males)	Median of 0.9 yrs (IQR 0.5–1.1); median at follow-up PSG of 2.1 yrs (1.5–2.6)	rhGH therapy, PSG (baseline PSG before age 2 yrs and follow-up PSG)	Median CAI at baseline: 6.6 (IQR 2.6, 12.1)/h improved to 2.3/h (*p* < 0.0001)	Initial GH dose 0.03 mg/kg/day	rhGH therapy initiated after baseline assessment in 19 infants; 10 infants with CSA at baseline started on rhGH therapy. Follow-up PSG revealed resolution of CSA in 7/10 infants	GH therapy did not appear to potentiate CSA
Scheermeyer et al. [26]	2017	Australia	Observational	31 infants (55% males); 42 toddlers (48% males)	Infants aged 2–12 mths; toddlers 13–24 mths	Low-dose rhGH in children (2–24 mths); PSG (before and within 6 mths after GH therapy initiated)	Mild to severe central and/or OSA in 40% of children prior to rhGH; onset or worsening of OSA in 2 infants in first mths and in 6 after 6–24 mths.	4.5 mg/m^2^/wk	rhGH therapy discontinued in 8 (11%) children due to increase in OSA in first 24 mths of therapy; severe OSA developed in first mths in 2 children	Possible increased risk of OSA during first wks of therapy
Lecka-Ambroziak et al. [45]	2017	Poland	Observational	36	Group 1: before rhGH 3.0 ± 3.0 yrs; Group 1a: after short-term rhGH 2.5 ± 0.8) yrs; Group 2: on rhGH 8.8 ± 5.1 yrs; Group 3: without rhGH 13.1 ± 4.4 yrs (severely obese)	rhGH therapy, PSG before (Group 1) and after initiating rhGH (Group 1a), on rhGH therapy for 4 ± 3.0 years (Group 2) and patients not treated due to severe obesity (Group 3)	OSA, AHI (/h): Group 1: 10.2 (±6.9); Group 1a: 12.0 (±5.8); Group 2: 9.0 (±6.5); Group 3: 8.2 (±5.4)	0.019 mg/kg/day (±0.006)	No difference in AHI before and after initiation of rhGH therapy between patients on rhGH therapy and untreated severely obese patients	Did not validate the hypothesis that initiation of rhGH therapy worsens OSA due to growth of lymphoid tissue; limitation: wide range in patient age
Zimmermann et al. [8]	2020	Germany	Longitudinal	62 (31 males); 21 initiated GH-therapy during and 41 after first year of life	0–2.5 yrs at baseline Group A (21 children): initiated GH-therapy during and Group B (41 children): after first year of life	rhGH, overnight respiratory polygraphy (before therapy (t0), after 3 (t1), 6 (t2) mths, and 1.2 (t3), 2.2 (t4), and 3.2 yrs (t5) after GH therapy initiated	Group A (5/21, 23.8%) patients versus Group B (15/41, 36.6%) patients with obstructive sleep apnea (OAHI ≥ 1.5)	0.028 mg/kg/day (range of 0.000–0.037)	No significant differences in OSA and CA, regardless of age at initiation of rhGH therapy; OSA increased during first 3 mths of therapy but decreased after 1 year; ODI changed during rhGH therapy	Increased incidence of severe OSA from baseline to 6 mths after GH therapy initiated
Caudri et al. [36]	2022	Australia	Multicenter	112	Median of 1.9 yrs (range of 0.1–13.5) at start of GH therapy	PSG before and after rhGH initiated	PSG (*n* = 94) included in analysis prior to initiating GH; median obstructive AHI 0.40 (range 0–4.9)/h, PSG after rhGH therapy 0.50 (0–51.7)/h; *p* = 0.13	NA	Worsening of OSA severity in 13% of children	Early identification of worsening OSA may prevent severe sequelae in subset of children; PSG should be performed after initiation of rhGH therapy to monitor for worsening OSA
Schaefer et al. [32]	2022	Australia	Retrospective single-center chart review	17 (8 males) on rhGH	Median of 11.6 yrs (range of 6.6–16.1)	rhGH, PSG (routine PSG pre- or post-GH therapy)	Total RDI/h: 9.8 (3.9–14.8); central AHI 4.2 (1.9–11.1)/h)	9 (53%) children on rhGH therapy at time of index PSG, and on therapy for median of 6.8 yrs (IQR 3.2–8.0)	15 (88%) had SDB, including CSA (*n* = 3, 18%), OSA (*n* = 4, 24%), both OSA and CSA (*n* = 5, 29%)	SDBs frequently recorded, also in patients on long-term rhGH therapy
Tan et al. [33]	2022	Canada	Retrospective	29 (41% males); 87 controls (46% males)	PWS, 4.4 ± 5.2 yrs; controls 4.4 ± 5.1 yrs	rhGH, 24 (82%) patients underwent PSG prior to rhGH; 12 before and after starting rhGH	AHI: PWS: 7.3 (IQR 11.8)/h; controls 6.0 (IQR 13.8); CI 2.9 (IQR 10.1)/h in patients with PWS vs. controls 1.8 (5.4)/h	rhGH therapy in 6 (21%) children	24/29 (82%) children with PWS underwent PSG prior to initiating rhGH therapy. No change in PSG parameters at 6.8 mths (95% CI 2.0, 11.7) after starting rhGH with a mean difference of 1.0 yrs (95% CI 1.3, 0.80) between PSG	Increases and decreases in respiratory events recorded after initiation of rhGH therapy, which did not affect respiratory parameters

Legend: AHI, apnea hypopnea index; A&T, adenotonsillectomy; CA, central apnea; CSA, central sleep apnea; ENT, ear, nose, and throat evaluation; GH, growth hormone; mths, months; OA, obstructive apnea; OAHI, obstructive apnea hypopnea index; OSA, obstructive sleep apnea; PSG, polysomnography; PWS, Prader–Willi syndrome; RCT, randomized controlled trial; rhGH, recombinant human GH; SA, sleep apnea; SDB, sleep disordered breathing; wks, weeks; yrs, years.

## 4. Discussion

The effects of GH therapy on the sleep of patients with PWS are not fully understood. Some studies have shown that GH therapy can improve sleep quality, while others have found that it can worsen sleep apnea. Its effects on sleep may depend on several factors, including the dosage and duration of therapy, underlying conditions, and individual variations in response to treatment. Close monitoring of respiratory function during sleep is essential in patients with PWS who are receiving GH therapy, as it may temporarily increase the risk of OSA in the early weeks of treatment and even worsen SDB in some patients. The severity of SDB in patients with PWS can vary considerably, even among patients receiving the same treatment regimen. Studies have shown that GH therapy can increase lymphoid tissue, partially obstructing the airways and making breathing more difficult during sleep. OSA severity did not appear to depend on age or degree of obesity in patients with PWS. Overall, the evidence on the effects of GH therapy on sleep in patients with PWS is mixed. 

### 4.1. GH Administration and Sleep-Disordered Breathing

In their randomized controlled trial (RCT) investigating the effects of GH therapy on lung function, sleep, behavior, and cognition in children with PWS [25], Haqq et al. found notable improvement in lung function and sleep quality, with improved spirometric parameters and a trend towards fewer occurrences and shorter duration of hypopnea and apnea events after rhGH therapy [25]. They went on to conclude that GH therapy improved body composition and resting energy expenditure (REE), thus potentially enhancing sleep quality and lung function. In contrast, Tan et al. [33] found that PWS was not associated with an altered risk of OSA and suggested that the risk and severity of OSA were similar for the patients with PWS and the controls. Interestingly, uninterrupted sleep and sleep efficiency (percentage of time spent sleeping compared to total time in bed) were better in the children with PWS than the controls.

The results of studies investigating the effects of GH therapy on SDB in patients with PWS are contradictory, with some indicating worsening sleep disturbances and others showing no involvement. The worsening of respiratory parameters during GH therapy has been observed or suggested by numerous studies [41]. Furthermore, Craig et al. [41] reported a higher mortality risk in children undergoing GH therapy in the first 9 months of therapy. Other studies have suggested that GH therapy has no impact on SDB [41]. Finally, three studies suggested a possible improvement in nocturnal respiratory disorder [25].

Given that sleep disturbances are prevalent in patients with PWS and that GH therapy does not significantly improve respiratory function [35], no definitive conclusions about its effect on sleep disturbances in patients with PWS can be drawn. Some evidence suggests that the initial risk of worsening SDB may improve in long-term therapy. Accordingly, GH therapy should be closely monitored, especially in patients with PWS, to identify its effects on respiratory function during sleep and sleep quality.

GH therapy was found to improve the AHI in 68% of patients with PWS, but 32% experienced worsening of SDB and obstructive events worsened in six patients during GH therapy. After 6 weeks of GH therapy, the obstructive AHI (oAHI) increased in 50% of patients with PWS, but the change was not statistically significant. GH therapy did not cause upper-airway obstruction or reduce upper-airway patency in patients with PWS [28]. 

Three studies yielded insight into the relationship between GH therapy and sleep. In individuals with PWS compared to healthy subjects, GH therapy improved hypopnea and apnea events after GH therapy [25]. In a group of patients receiving GH therapy, obstructive sleep apnea (oAHI ≥ 1.5) was noted in 23.8% of Group A and in 36.6% of Group B. OSA increased during the first three months of therapy but decreased after one year. Changes in the ODI were observed during GH therapy [8]. The pre-treatment PSG showed a median oAHI of 0.40 (range of 0–4.9), and in the post-treatment PSG, the median oAHI increased to 0.50 (0–51.7). Worsening OSA severity was observed in 13% of children. The study suggested that early identification of worsening OSA by PSG after the initiation of GH therapy may help prevent severe complications in a subset of children [36]. 

Moreover, long-term GH therapy can lead to adenotonsillar hypertrophy and exacerbate nighttime apnea in children with PWS [43]. Early diagnosis and sleep monitoring by PSG are necessary to monitor for SDB [46]. Adenotonsillectomy may be effective in most children with mild to moderate OSA and PWS, but it may not be curative in children with severe OSA [28,42]. Close monitoring of respiratory function during sleep is essential before initiating GH therapy [23], as it may temporarily increase the risk of OSA in the early weeks of treatment and even worsen SDB in some patients [23]. While some patients may experience fewer AHI events and improved SpO_2_ during sleep after GH therapy, the potential benefits must be carefully weighed against the risks of prescribing GH. Also, it is essential to assess factors such as adenotonsillar hypertrophy and other factors that may alter respiratory function during sleep before and during GH therapy [43].

### 4.2. Influencing Variables

Common differences among the studies regarding the type of GH therapy regard the dosage and treatment duration. Some studies combined GH therapy with other interventions, such as PSG, anthropometric evaluation, or OSA surgery, or applied various assessment parameters, such as PSG, multiple sleep latency test (MSLT), anthropometric evaluation, or gas exchange data. The differences in results suggest that SDB severity in patients with PWS can vary considerably across studies. Such differences may depend on various factors, including the sample population, method of SDB assessment, and GH therapy duration and effectiveness. For example, Haqq et al. [25] found no significant differences in apnea and hypopnea events between patients with PWS and healthy subjects. Caudri et al. [36] reported no significant differences in AHI before and after the start of GH therapy. Miller et al. [36] demonstrated that GH therapy improved the AHI by an average of 1.2/h and the number of central apneas (CA) by 1.7/h. Salvatoni et al. [28] found that the AHI was increased in 50% of patients and decreased in 31% of patients. Meyer et al. [42] reported that 69% of patients had mild to moderate obstructive sleep apnea. Vandeleur et al. (2013) diagnosed OSA in 44% of patients [23]. Zimmermann et al. [8] found that 23.8% of patients in Group A and 36.6% in Group B had OSA. The variability in rhGH dosage can be due to physician preferences, differences in treatment guidelines, or specific patient characteristics. Since different dosages may impact the results and effectiveness of GH therapy in managing SDB in patients with PWS, further studies in this area should include a broader range of variables and more patients to better understand the relationship between GH therapy and sleep improvement in patients with PWS.

Several variables should be considered when examining the impact of GH therapy on sleep patterns. First, GH therapy can potentially influence the timing and secretion pattern of GH. External administration increases the body’s GH levels, potentially disrupting the natural secretion rhythm [47,48]. This may lead to changes in the timing and duration of deep sleep stages, during which GH is primarily released [49]. Second, GH therapy can affect sleep quality. Increased GH levels may enhance sleep efficiency and facilitate restorative sleep through improvement in slow-wave sleep, which plays a crucial role in physical recovery and growth, and enhancement in REM sleep, which is essential for cognitive functioning and emotional regulation. 

The impact of GH therapy on sleep characteristics can vary from person to person. Factors such as the GH dosage and duration of treatment, underlying conditions, and individual variation in response to treatment can all contribute to the differences in effects. Finally, recent studies have shown that recombinant human growth hormone (rhGH) can help to improve muscle tone and reduce body fat in children with PWS. Children with PWS are typically noted to have increased fat mass and decreased lean body mass [50]. Muscle mass is reduced by 25–37% in children with PWS, which can contribute to weakness and hypotonia [51]. rhGH therapy can improve body composition in children with PWS, increasing muscle bulk and reducing body fat, and possibly encouraging greater physical activity [52]. rhGH therapy can also impact the respiratory status of children with PWS. Some studies have shown that rhGH therapy increased lymphoid tissue, which can partially obstruct the airways and make breathing more difficult during sleep [52]. OSA severity did not appear to depend on age or degree of obesity. This suggests that other mechanisms come into play, probably hypotonia and/or facial dysmorphic features, in the consistency of OSA throughout a person’s life span [38]. 

### 4.3. Study Limitations

The relationship between GH therapy and sleep in patients with PWS may depend on several variables. We found marked variability across studies in the authors’ countries of origin, including the United States, the Netherlands, Australia, Canada, Italy, Sweden, Switzerland, and Germany. Within the context of the present review, variability related to the countries of origin could impact the results in different ways, such as the clinical approach, healthcare resources, study protocols, and study population characteristics. The variability in the results related to the study design may reflect differences in the data collection, methodology, internal and external validity of the study, and conclusions. 

The variability regarding the number of patients (sample size) can affect a study’s representativeness and statistical power. Studies with few participants may have limited statistical ability to identify significant differences among groups. 

Out of a total of 23 studies, 22 conducted PSG, 2 of which conducted overnight respiratory polygraphy [8,38], while 1 study did not state whether PSG was performed [39], and 13 studies performed follow-up PSG [8,25,26,27,31,32,33,34,36,37,41,43,45]. A total of 1242 patients with PWS were involved in 22 studies, with a minimum age of 2 months [29] and a maximum age of 63 years [35] in 23 studies. The study protocol designs were as follows: two RCTs [25,31], three longitudinal [8,28,34], 1 prospective [41], seven observational [23,30,37,43,44,45], one pilot [29], four retrospective [24,27,32], one comparative [42], one clinical case series [35], one clinical trial [39], and two multicentric [36,38]. The total number of subjects was 1294, the maximum sample size was 328 patients [41], and the minimum was 5 [27]. 

Differences in patient age can influence study results in various ways, such as treatment response and PWS characteristics (patient age may reflect different stages of PWS). Age categories vary across studies and are not always explicitly defined: neonates/infants [29,37], prepubertal [23,31,39], children [33,42,43,45], patients [24,27,30,36] or non-severely obese [28]. In addition, one study administered rhGH therapy or placebo for 6 months, then an alternative intervention for 6 months [25], one study administered rhGH at baseline and after 6 weeks [29], four studies administered rhGH and PSG [8,29,35], six studies administered rhGH [26,27,32,41,42,44], and one study administered rhGH, PSG, and the Multiple Sleep Latency Test (MSLT) [30]. Some studies reported specific dosages, such as 0.043 mg/kg/day [25], 0.24 mg/kg/wk [29], 1 mg/m^2^/day [31], 0.23–0.22 mg/kg/wk [41], 0.22 mg/kg/wk [30], 1 mg/m^2^/day [29], 0.25 mg/kg/wk [24], 0.03 mg/kg/day [28], 0.010–0.030 mg/kg/day [43], and 0.028 mg/kg/day [8], while others gave no information [23,27,32,33,35,36,38,42,44].

## 5. Conclusions

The effects of rhGH therapy on SDB in children with PWS remain debated. Some studies have reported that rhGH therapy may worsen respiratory parameters, while others have found no significant effect on SDB. Therefore, it is advisable to thoroughly evaluate its effect on respiratory function during sleep and to perform PSG before initiating rhGH therapy to determine whether there is a need for adenotonsillectomy before starting therapy. Adenotonsillectomy should be performed before starting GH therapy in cases of sleep apnea. This will help to minimize the risk of respiratory complications during treatment. Close monitoring of sleep disorders is essential in patients with PWS receiving rhGH therapy to ensure effective and safe treatment. Further research is needed to better understand the relationship between GH therapy and SDB in patients with PWS and identify the factors influencing treatment outcomes.

## Data Availability

Data sharing is not applicable to this article.

## References

[1] Schwartz J.R., Roth T. (2008). Neurophysiology of sleep and wakefulness: Basic science and clinical implications. Curr. Neuropharmacol..

[2] Alrousan G., Hassan A., Pillai A.A., Atrooz F., Salim S. (2022). Early Life Sleep Deprivation and Brain Development: Insights From Human and Animal Studies. Front. Neurosci..

[3] Vgontzas A.N., Mastorakos G., Bixler E.O., Kales A., Gold P.W., Chrousos G.P. (1999). Sleep deprivation effects on the activity of the hypothalamic-pituitary-adrenal and growth axes: Potential clinical implications. Clin. Endocrinol..

[4] Iughetti L., Tornese G., Street M.E., Napoli F., Giavoli C., Antoniazzi F., Stagi S., Luongo C., Azzolini S., Ragusa L. (2016). Long-term safety and efficacy of Omnitrope®, a somatropin biosimilar, in children requiring growth hormone treatment: Italian interim analysis of the PATRO Children study. Ital. J. Pediatr..

[5] Grimberg A., DiVall S.A., Polychronakos C., Allen D.B., Cohen L.E., Quintos J.B., Rossi W.C., Feudtner C., Murad M.H. (2016). Guidelines for Growth Hormone and Insulin-Like Growth Factor-I Treatment in Children and Adolescents: Growth Hormone Deficiency, Idiopathic Short Stature, and Primary Insulin-Like Growth Factor-I Deficiency. Horm. Res. Paediatr..

[6] Antoniazzi F., Cavarzere P., Gaudino R. (2015). Growth hormone and early treatment. Minerva Endocrinol..

[7] Cavarzere P., Gaudino R., Sandri M., Ramaroli D.A., Pietrobelli A., Zaffanello M., Guzzo A., Salvagno G.L., Piacentini G., Antoniazzi F. (2020). Growth hormone retesting during puberty: A cohort study. Eur. J. Endocrinol..

[8] Zimmermann M., Laemmer C., Woelfle J., Fimmers R., Gohlke B. (2020). Sleep-Disordered Breathing in Children with Prader-Willi Syndrome in Relation to Growth Hormone Therapy Onset. Horm. Res. Paediatr..

[9] Goldstone A.P., Holland A.J., Hauffa B.P., Hokken-Koelega A.C., Tauber M. (2008). Recommendations for the diagnosis and management of Prader-Willi syndrome. J. Clin. Endocrinol. Metab..

[10] Deal C.L., Tony M., Höybye C., Allen D.B., Tauber M., Christiansen J.S. (2013). GrowthHormone Research Society workshop summary: Consensus guidelines for recombinant human growth hormone therapy in Prader-Willi syndrome. J. Clin. Endocrinol. Metab..

[11] Lyons M.M., Bhatt N.Y., Pack A.I., Magalang U.J. (2020). Global burden of sleep-disordered breathing and its implications. Respirology.

[12] Zaffanello M., Lippi G., Arman N., Piazza M., Tenero L., Piacentini G. (2019). Popularity of sleep disordered breathing in childhood: An analysis of worldwide search using Google Trends. Transl. Pediatr..

[13] Zaffanello M., Ferrante G., Zoccante L., Ciceri M.L., Nosetti L., Tenero L., Piazza M., Piacentini G. (2023). Predictive Power of Oxygen Desaturation Index (ODI) and Apnea-Hypopnea Index (AHI) in Detecting Long-Term Neurocognitive and Psychosocial Outcomes of Sleep-Disordered Breathing in Children: A Questionnaire-Based Study. J. Clin. Med..

[14] Zaffanello M., Piacentini G., La Grutta S. (2020). Beyond the growth delay in children with sleep-related breathing disorders: A systematic review. Panminerva Med..

[15] Lagravère M.O., Zecca P.A., Caprioglio A., Fastuca R. (2019). Metabolic effects of treatment in patients with obstructive sleep apnea: A systematic review. Minerva Pediatr..

[16] Tagetti A., Bonafini S., Zaffanello M., Benetti M.V., Vedove F.D., Gasperi E., Cavarzere P., Gaudino R., Piacentini G., Minuz P. (2016). Sleep-disordered breathing is associated with blood pressure and carotid arterial stiffness in obese children. J. Hypertens..

[17] Lo Bue A., Salvaggio A., Insalaco G. (2020). Obstructive sleep apnea in developmental age. A narrative review. Eur. J. Pediatr..

[18] Brockmann P.E., Gozal D. (2022). Neurocognitive Consequences in Children with Sleep Disordered Breathing: Who Is at Risk?. Children.

[19] Todd C.A., Bareiss A.K., McCoul E.D., Rodriguez K.H. (2017). Adenotonsillectomy for Obstructive Sleep Apnea and Quality of Life: Systematic Review and Meta-analysis. Otolaryngol.–Head. Neck Surg. Off. J. Am. Acad. Otolaryngol.-Head. Neck Surg..

[20] Zaffanello M., Antoniazzi F., Tenero L., Nosetti L., Piazza M., Piacentini G. (2018). Sleep-disordered breathing in paediatric setting: Existing and upcoming of the genetic disorders. Ann. Transl. Med..

[21] Gerard J.M., Garibaldi L., Myers S.E., Aceto T., Kotagal S., Gibbons V.P., Stith J., Weber C. (1997). Sleep apnea in patients receiving growth hormone. Clin. Pediatr..

[22] Mistretta A., Modica D.M., Pitruzzella A., Burgio S., Lorusso F., Billone S., Valenti C., Vita G., Poma S., Amata M. (2022). OSAHS Growth Impairment and Resolution after Adenotonsillectomy in Children. Iran. J. Otorhinolaryngol..

[23] Vandeleur M., Davey M.J., Nixon G.M. (2013). Are sleep studies helpful in children with Prader-Willi syndrome prior to commencement of growth hormone therapy?. J. Paediatr. Child. Health.

[24] Fillion M., Deal C., Van Vliet G. (2009). Retrospective study of the potential benefits and adverse events during growth hormone treatment in children with Prader-Willi syndrome. J. Pediatr..

[25] Haqq A.M., Stadler D.D., Jackson R.H., Rosenfeld R.G., Purnell J.Q., LaFranchi S.H. (2003). Effects of growth hormone on pulmonary function, sleep quality, behavior, cognition, growth velocity, body composition, and resting energy expenditure in Prader-Willi syndrome. J. Clin. Endocrinol. Metab..

[26] Scheermeyer E., Harris M., Hughes I., Crock P.A., Ambler G., Verge C.F., Bergman P., Werther G., Craig M.E., Choong C.S. (2017). Low dose growth hormone treatment in infants and toddlers with Prader-Willi syndrome is comparable to higher dosage regimens. Growth Horm. IGF Res..

[27] DeMarcantonio M.A., Darrow D.H., Gyuricsko E., Derkay C.S. (2010). Obstructive sleep disorders in Prader-Willi syndrome: The role of surgery and growth hormone. Int. J. Pediatr. Otorhinolaryngol..

[28] Salvatoni A., Veronelli E., Nosetti L., Berini J., de Simone S., Iughetti L., Bosio L., Chiumello G., Grugni G., Delu G. (2009). Short-term effects of growth hormone treatment on the upper airways of non severely obese children with Prader-Willi syndrome. J. Endocrinol. Investig..

[29] Miller J.L., Shuster J., Theriaque D., Driscoll D.J., Wagner M. (2009). Sleep disordered breathing in infants with Prader-Willi syndrome during the first 6 weeks of growth hormone therapy: A pilot study. J. Clin. Sleep. Med..

[30] Williams K., Scheimann A., Sutton V., Hayslett E., Glaze D.G. (2008). Sleepiness and sleep disordered breathing in Prader-Willi syndrome: Relationship to genotype, growth hormone therapy, and body composition. J. Clin. Sleep. Med..

[31] Festen D.A., de Weerd A.W., van den Bossche R.A., Joosten K., Hoeve H., Hokken-Koelega A.C. (2006). Sleep-related breathing disorders in prepubertal children with Prader-Willi syndrome and effects of growth hormone treatment. J. Clin. Endocrinol. Metab..

[32] Schaefer J., Davey M.J., Nixon G.M. (2022). Sleep-disordered breathing in school-aged children with Prader-Willi syndrome. J. Clin. Sleep. Med..

[33] Tan Q., He X.T.T., Kang S., Haqq A.M., MacLean J.E. (2022). Preserved Sleep for the Same Level of Respiratory Disturbance in Children with Prader-Willi Syndrome. Int. J. Mol. Sci..

[34] Miller J., Silverstein J., Shuster J., Driscoll D.J., Wagner M. (2006). Short-term effects of growth hormone on sleep abnormalities in Prader-Willi syndrome. J. Clin. Endocrinol. Metab..

[35] Katz-Salamon M., Lindgren A.C., Cohen G. (2012). The effect of growth hormone on sleep-related cardio-respiratory control in Prader-Willi syndrome. Acta Paediatr. Int. J. Paediatr..

[36] Caudri D., Nixon G.M., Nielsen A., Mai L., Hafekost C.R., Kapur N., Seton C., Tai A., Blecher G., Ambler G. (2022). Sleep-disordered breathing in Australian children with Prader-Willi syndrome following initiation of growth hormone therapy. J. Paediatr. Child. Health.

[37] Khayat A., Narang I., Bin-Hasan S., Amin R., Al-Saleh S. (2017). Longitudinal evaluation of sleep disordered breathing in infants with Prader-Willi syndrome. Arch. Dis. Child..

[38] Pavone M., Caldarelli V., Khirani S., Colella M., Ramirez A., Aubertin G., Crinò A., Brioude F., Gastaud F., Beydon N. (2015). Sleep disordered breathing in patients with Prader-Willi syndrome: A multicenter study. Pediatr. Pulmonol..

[39] Meinhardt U., Christiansen J.S., Farholt S., Lammer C., Ostergaard J.R., Schmidt F., Kappelgaard A.M., Eiholzer U. (2013). The efficacy and safety of long-term Norditropin(R) treatment in children with Prader-Willi syndrome. Horm. Metab. Res..

[40] Lecka-Ambroziak A., Wysocka-Mincewicz M., Świercz A., Jędrzejczak M., Szalecki M. (2021). Comparison of Frequency and Severity of Sleep-Related Breathing Disorders in Children with Simple Obesity and Paediatric Patients with Prader-Willi Syndrome. J. Pers. Med..

[41] Craig M.E., Cowell C.T., Larsson P., Zipf W.B., Reiter E.O., Albertsson Wikland K., Ranke M.B., Price D.A. (2006). Growth hormone treatment and adverse events in Prader-Willi syndrome: Data from KIGS (the Pfizer International Growth Database). Clin. Endocrinol..

[42] Meyer S.L., Splaingard M., Repaske D.R., Zipf W., Atkins J., Jatana K. (2012). Outcomes of adenotonsillectomy in patients with Prader-Willi syndrome. Arch. Otolaryngol. Head. Neck Surg..

[43] Berini J., Spica Russotto V., Castelnuovo P., Di Candia S., Gargantini L., Grugni G., Iughetti L., Nespoli L., Nosetti L., Padoan G. (2013). Growth hormone therapy and respiratory disorders: Long-term follow-up in PWS children. J. Clin. Endocrinol. Metab..

[44] Cohen M., Hamilton J., Narang I. (2014). Clinically important age-related differences in sleep related disordered breathing in infants and children with Prader-Willi Syndrome. PLoS ONE.

[45] Lecka-Ambroziak A., Jedrzejczak M., Wysocka-Mincewicz M., Szalecki M. (2017). Sleep-related breathing disorders in patients with Prader-Willi syndrome depending on the period of growth hormone treatment. Endokrynol. Pol..

[46] Stafler P., Wallis C. (2008). Prader-Willi syndrome: Who can have growth hormone?. Arch. Dis. Child..

[47] Garnier P., Raynaud F., Job J.C. (1988). Growth hormone secretion during sleep. I. Comparison with GH responses to conventional pharmacologic stimuli in pubertal and early pubertal short subjects. Effects of treatment with human GH in patients with discrepant measurements of GH secretion. Horm. Res..

[48] Morris C.J., Aeschbach D., Scheer F.A. (2012). Circadian system, sleep and endocrinology. Mol. Cell Endocrinol..

[49] Smith P.C., Mong J.A. (2019). Neuroendocrine Control of Sleep. Curr. Top. Behav. Neurosci..

[50] Carrel A.L., Myers S.E., Whitman B.Y., Eickhoff J., Allen D.B. (2010). Long-term growth hormone therapy changes the natural history of body composition and motor function in children with prader-willi syndrome. J. Clin. Endocrinol. Metab..

[51] Reus L., Zwarts M., van Vlimmeren L.A., Willemsen M.A., Otten B.J., Nijhuis-van der Sanden M.W. (2011). Motor problems in Prader-Willi syndrome: A systematic review on body composition and neuromuscular functioning. Neurosci. Biobehav. Rev..

[52] Bridges N. (2014). What is the value of growth hormone therapy in Prader Willi syndrome?. Arch. Dis. Child..

